# Environmental and biotic drivers of *Aedes albopictus* spatiotemporal distribution in a subtropical city: A major transit hub on Argentina’s triple border

**DOI:** 10.1371/journal.pntd.0013989

**Published:** 2026-07-28

**Authors:** Julieta A. Siches, Maria V. Micieli, Pablo E. Berrozpe, Maria del Rosario Iglesias, Juan J. García, María V. Cardo

**Affiliations:** 1 Ministerio de Salud de la Nación Argentina - Administración Nacional de Laboratorios e Institutos de la Salud (“Dr. Carlos Malbrán”), Ciudad Autónoma de Buenos Aires, Argentina; 2 Universidad Nacional de La Plata - Consejo Nacional de Investigaciones Científicas y Técnicas - Centro de Estudios Parasitológicos y de Vectores (CEPAVE), La Plata, Buenos Aires, Argentina; 3 Consejo Nacional de Investigaciones Científicas y Técnicas, Buenos Aires, Argentina; 4 Administración Nacional de Laboratorios e Institutos de la Salud (“Dr. Carlos Malbrán”) - Instituto Nacional de Parasitología (“Dr. Fatala Chabén”), Ciudad Autónoma de Buenos Aires, Argentina; 5 Administración de Parques Nacionales - Dirección Nacional de Conservación, Ciudad Autónoma de Buenos Aires, Argentina; 6 Universidad Nacional de Misiones - Facultad de Ciencias Forestales, Eldorado, Misiones, Argentina; 7 Universidad Nacional de San Martin – Consejo Nacional de Investigaciones Científicas y Tecnológicas - Instituto de Investigación e Ingeniería Ambiental, Escuela de Hábitat y Sostenibilidad, Campus Miguelete, San Martín, Provincia de Buenos Aires, Argentina (IIIA-UNSAM-CONICET), San Martín, Buenos Aires Argentina; Centers for Disease Control and Prevention, UNITED STATES OF AMERICA

## Abstract

Biophysical and ecological factors are known to shape the distribution of mosquito vectors, yet their interaction across heterogeneous and rapidly transforming landscapes remains insufficiently understood. We assessed the occurrence of *Aedes albopictus* in relation to environmental and biotic drivers in the Iguazú Department, Misiones Province, Argentina, within the tri-border region shared with Brazil and Paraguay, a key epidemiological corridor for arboviral transmission. Mosquitoes were monitored using adult activity sensors (ovitraps) across 81 sites representing urban, periurban, and wild environments (later divided in pristine and disturbed wild) in eleven sampling sessions between April 2019 and February 2020. Environmental characterization integrated field data and remotely sensed biophysical variables at different spatial scales relevant to vector ecology, which were included in generalized linear mixed models together with the presence of *Ae. aegypti* as covariate to account for potential interspecific effects, and co-occurrence of both *Aedes* species was further evaluated using Bayesian analyses. *Aedes albopictus* showed a strongly stratified spatial pattern, with 30.4-30.6% positive ovitraps in periurban and disturbed wild, in comparison with 14.2% for urban and only 3.9% for pristine wild. Occurrence probability increased with intermediate levels of monthly precipitation and minimum temperature, showed nonlinear responses to vegetation cover, and decreased with impervious surfaces, explaining 67% of the variability. Co-occurrence analyses indicated positive associations in urban and periurban environments, suggesting partial niche overlap under anthropogenic conditions. Our results identify periurban and disturbed wild environments as critical ecological interfaces structuring *Ae. albopictus* distribution. These findings provide a robust basis for targeted surveillance and highlight the need for coordinated, cross-border vector management strategies in rapidly transforming subtropical regions.

## Author summary

Mosquitoes that transmit diseases are highly sensitive to their surroundings, yet we know little about how they respond to landscapes being rapidly transformed by human activities, especially in border regions where people and pathogens move frequently. In this study, we focused on *Aedes albopictus*, a mosquito capable of transmitting dengue and other arboviruses, in Puerto Iguazú, a subtropical city in Argentina located near the borders with Brazil and Paraguay. We monitored its presence monthly across urban, periurban, and natural environments for nearly one year. *Ae. albopictus* was most common in periurban areas where the city meets the countryside and in disturbed zones within protected areas such as trails and visitor facilities. In contrast, it was rare in highly urbanized centers with many paved surfaces and almost absent from pristine, undisturbed forests. Its occurrence was linked to rainfall, temperature, and vegetation, and it often co-occurred with *Ae. aegypti,* another important disease vector, especially in human modified settings. These findings reveal that transitional landscapes, neither fully urban nor completely wild, act as critical hotspots for this mosquito. For public health, this means that surveillance and control efforts should focus on these interface zones, and that environmental land use planning could become a key tool for preventing vector borne diseases in rapidly changing subtropical regions.

## Introduction

Over the past five decades, arboviral diseases —such as dengue, Zika, yellow fever and chikungunya— have emerged as some of the most widespread and pressing public health challenges in the Americas. These infections have been particularly burdensome in tropical and subtropical regions, where they account for the highest rates of morbidity and mortality [1]. Transmission of these vector-borne diseases depends on the simultaneous presence of the host, the vector, and the pathogen within the same geographic area [2]. Moreover, transmission dynamics are influenced by the diversity, abundance, and spatial distribution of both hosts and vectors within the landscape, resulting in micro-spatial heterogeneity in transmission patterns [3]. Understanding the ecological characteristics of disease vectors is therefore crucial for identifying their distribution patterns and for developing more effective strategies to address current and emerging epidemiological threats.

*Aedes albopictus* (Diptera: Culicidae, Stegomyia) is an invasive species native to Southeast Asia, recognized as a competent vector for a wide range of arboviruses. These include the four dengue virus serotypes as well as viruses responsible for Japanese encephalitis, Potosi, Keystone, Tensaw, eastern equine encephalitis, yellow fever, chikungunya and Zika [4,5]. Notably, the potential role of *Ae. albopictus* in chikungunya virus transmission has gained particular attention in the American continent, where it has been shown to be a more effective vector than *Ae. aegypti* [6]. This is specially concerning in regions where its geographic range has recently expanded, such as parts of Europe [7].

The most likely pathway for the introduction of *Ae. albopictus* into the Americas is through the international trade of used vehicle tires with Southeast Asia. In 1985, the species was first recorded in Harris County, Texas, United States [8]. Within just two years, it had been reported in 15 of the 50 US states and by 1997, its distribution had extended to 25 states [9]. In South America, the first report occurred in Brazil in 1986 [10] and, by 2002, it had been detected in 20 of the 27 Brazilian states [11].

In Argentina, *Ae. albopictus* was first recorded in 1998 in San Antonio, Misiones Province, located in the northeast of the country [12]. Since then, its presence has been confirmed in a few additional localities within Misiones Province and a small area of neighboring Corrientes Province, with the current distribution extending between latitudes 25° 28’ and 28° 10’ S. In other words, twenty-five years after its first detection in Argentina, the distribution of *Ae. albopictus* remains limited, with only a few locations showing signs of stabilization [13,14]. Although several studies have explored potential factors restricting its distribution, including competitive exclusion, the composition of container-breeding mosquito communities, environmental preferences, and egg diapause [13,15], the reasons behind the failure of *Ae. albopictus* to expand in Argentina remain uncertain.

Health risks linked to biodiversity loss and ecosystem degradation have been repeatedly highlighted, such as the increased burden of vector-borne diseases resulting from land degradation and land cover transformation [16]. For instance, in South America, land-use changes such as road construction have been strongly associated with increased incidence of malaria, leishmaniasis, and other parasitic diseases among workers [17]. Development projects can increase human exposure to natural mosquito habitats. On the other hand, they can also create more suitable habitats for the proliferation of these insects, such as forest edges and associated microclimates [18]. The conversion of wild forests into agricultural land, in particular, has been shown to exacerbate the spread of mosquito-borne diseases [17].

Numerous studies suggest that *Ae. albopictus* population densities fluctuate interannually in response to climatic variability [19]. For example, the highest abundances have been observed at temperatures around 25 °C [20], while conditions simulating unfavorable winter scenarios such as short photoperiods (8 h light: 16 h dark) and temperatures of approximately 16 ± 2.3 °C have been shown to inhibit completion of its life cycle [21]. Adult *Ae. albopictus* abundance has also been positively correlated with the density of larval habitats, which is in turn influenced by microclimatic conditions [20]. Precipitation plays a key role by increasing the number of outdoor containers that accumulate rainwater, thereby creating suitable oviposition sites. Vegetation indices are frequently used in mosquito studies; specifically, the Normalized Difference Vegetation Index (NDVI) has been consistently associated with infestation levels of both immature and adult *Ae. albopictus* [15,19]. *Ae. albopictus* abundance has also been reported to be strongly associated with socio-ecological factors, including the accessibility and quality of public services, vegetation degradation, and the state of urban infrastructure [22].

For its part, *Ae. aegypti* (L.) is widely recognized as the primary vector of several major arboviral diseases in urban environments, both in the Americas and globally [23]. In Argentina, its distribution is broad and expanding, encompassing the entire northern and central regions of the country as well as reaching the northern limits of the Patagonian region [24]. Coexistence between *Ae. albopictus* and *Ae. aegypti* in the same larval containers has been reported [13], and in some contexts, *Ae. albopictus* has been hypothesized to competitively displace *Ae. aegypti* [25]. However, such displacement has not been observed in Argentina, where recent studies conversely indicate that *Ae. aegypti* may outcompete *Ae. albopictus* in urban environments or that the two species may exhibit co-dominance in rural settings [13]. Habitat segregation has been proposed as a mechanism facilitating coexistence by reducing direct competition between species [26]. In other regions, *Ae. aegypti* predominates in urban environments, whereas *Ae. albopictus* is more common in rural areas, with both species overlapping in periurban zones [27]. This segregation pattern could be attributed to a preference of *Ae. albopictus* for environments with certain characteristics, such as the availability of particular breeding sites, a greater diversity of food sources, and a higher number of shelters, rather than solely by competitive exclusion. This is especially relevant considering that *Ae. albopictus* exhibits a more peridomiciliary behavior, in contrast to the intradomiciliary preference of *Ae. aegypti* [28].

The study area, Puerto Iguazú, is located in the northwesternmost part of Misiones Province, Argentina, at the tri-border area shared with Brazil and Paraguay. During recent years, the city has experienced rapid urban expansion characterized by uneven, disordered, and unplanned growth, resulting in limited access to essential public services such as potable water, sewage, electricity, and waste management. Iguazú National Park, located 17 km apart from Puerto Iguazú, is one of the most biodiverse protected areas in Argentina and boasts the highest number of endemic species in the country [29]. Despite its protected status, the park is impacted by human activity, including tourism infrastructure and housing for park staff. Managing and preserving this natural space poses major challenges, especially given the high volume of visitors [29]. These challenges include increased human–wildlife interactions, which elevates the risk of exposure to both known and emerging zoonotic diseases and management waste [30].

From an epidemiological perspective, the Iguazú Department holds strategic importance due to its high volume of human movement across land and river borders, as well as through its airport, which operates both domestic and international flights. The latter has been identified as a major contributor to the spread and intensification of arboviral diseases such as dengue [31]. Cases of both imported and autochthonous dengue have been consistently reported from the 2015/16 to the 2023/24 seasons, with the exception of the 2016/17 season [32]. Additionally, the 2022/23 season recorded a notable outbreak of chikungunya fever [33]. This underscores the Iguazú Department’s critical role as both a point of entry and establishment for circulating arboviruses and highlights the potential role of *Ae. albopictus* as a bridge vector between sylvatic transmission cycles and periurban/urban environments [34,35].

In this context, gaining insights into the ecology of disease vectors is essential for deciphering their distribution patterns and developing more effective strategies to address emerging epidemiological challenges. The objective of the present study was to assess the environmental and biotic factors associated with the spatiotemporal distribution of *Ae. albopictus* in northeastern Argentina, using the city of Puerto Iguazú and the Iguazú National Park as a study case. This study is guided by four main lines of inquiry, concerning the association between *Ae. albopictus* distribution and the degree of anthropogenic intervention, climatic factors, land cover class, and the presence of *Ae. aegypti*. Addressing these lines, will contribute to a deeper understanding of the determinants shaping the distribution of *Ae. albopictus*, and clarify the constraints on its expansion, with the aim to propose vector control and prevention tools for mitigating zoonoses transmission. This knowledge is particularly relevant in the context of recent climatic changes, intensified by global warming, which pose profound implications for the health of both human populations and ecosystems.

## Materials and methods

### Study area

**Fig 1:**
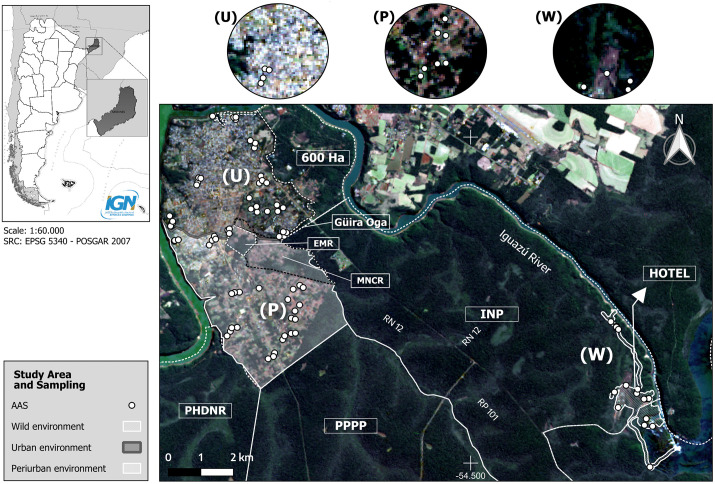
Study area. **Legend:** categorized a priori in three environmental types, urban **(U)**, periurban (P) and wild **(W)**, and surrounding natural areas, including: Puerto Peninsula Provincial Park (PPPP), the Peninsula Harbor Defense Nature Reserve (PHDNR), Iguazu National Park and Reserve (INP), Mboreré Municipal Natural and Cultural Reserve (MNCR), “El Eucaliptal” Municipal Natural Reserve (EMR), Andrés Giai Protected Landscape (Güira Oga), and the Iriapú Reserve (600 Ha). The primary routes and roads in the study area are RN 12 and RP 101. The distribution of adult activity sensors (AAS) is also indicated on the map (white dots). Administrative boundaries obtained from the Instituto Geográfico Nacional (IGN), Argentina (https://www.ign.gob.ar/sig). This Figure include Landsat 8 Operational Land Imager (OLI) imagery courtesy of the **U.**S. Geological Survey (USGS), available in the public domain (April 2019 – February 2020).

Puerto Iguazú is located in the Paranaense Forest ecoregion, which is part of the Atlantic Forest complex in Misiones Province, Argentina (Fig 1). This ecoregion is characterized by a semi-deciduous forest with differentiated tree strata, abundant epiphytes, bamboo, and lianas. The climate is subtropical, with an average annual temperature of 21 °C, ranging from 24 °C in summer to 14 °C in winter. Annual precipitation is approximately 1.800 mm, concentrated in the summer season [36].

### Entomology sampling

Based on the visual analysis of high-resolution satellite imagery and national sociodemographic data obtained from the official statistics and census institute [37–39], for sampling purposes the study area was classified a priori into three environmental types: urban (U), characterized by high population density and infrastructure, corresponding to the central area of Puerto Iguazú; periurban (P), defined by a mix of rural and urban elements with lower population density and subsistence agricultural activities; and wild (W), featuring predominant native vegetation and minimal anthropogenic intervention, encompassing the public use and trail areas of the Iguazú National Park (INP). A vector layer of polygons, composed of geometric shapes used to represent spatial features, was created on the image to delineate these environments (Fig 1). This classification was further evaluated by ordering the sampling units in a multivariate space as a function of the environmental variables recorded in a Principal Component Analysis for a posteriori classification (see below).

Sampling was conducted from April 2019 to February 2020 on a monthly basis, obtaining samples for all seasons of the year. Ovitraps consisting of 1 L black plastic containers lined with corrugated cardboard to support egg deposition were employed as adult activity sensors (AAS) to record the presence or absence of *Aedes* spp. (≥1 egg or larva per trap) [40]. The study area was divided into 400 m^2^ quadrants, which were randomly selected to cover 10% of the total area in each of the three defined environmental types. Four AAS were randomly placed in each selected quadrant, with the exception of quadrants U3, P7, P3 and W4, where three AAS were deployed, and W5, which had only one AAS, due to accessibility issues. Therefore, a total of 35, 30, and 16 AAS were deployed in U, P, and W, respectively. They were positioned at 1 -1.5 m height [41], filled to 50% of their capacity with dechlorinated tap water, and left uncovered for 6–8 days to allow oviposition. After this period, the AAS were covered (deactivated) until the next sampling cycle. Any missing or damaged AAS were replaced with new ones. After the last sampling event, the traps were dismantled.

The egg supports were transported to the insectary at the National Tropical medicine institute (INMeT, ANLIS Malbrán - MSAL) in sealed, properly labeled bags. Upon arrival, the supports were spread out and allowed to dry in an incubator at 19 ± 2 °C until immersion. The hatched larvae were reared in 250 ml plastic containers covered with semi-transparent polyester mesh that permitted air circulation and light exposure and fed a standard laboratory diet. Once the larvae reached the pupal stage, they were transferred to smaller containers (20 ml capacity), which were placed in emergence cages. The emerged adults were euthanized with acetone in a lethal chamber, dried, mounted using ad-hoc techniques, and identified under a stereoscopic microscope using dichotomous keys [42].

### Explanatory variables

#### Macrohabitat.

Spectral indices were used to characterize the land surface based on its spectral response, providing information on vegetation greenness, moisture, photosynthetic activity, and the presence of built-up areas or bare soil. For each season -defined as Autumn (April-June), Winter (July-September), Spring (October-December), and Summer (January-February)- a preliminary processing stage was conducted using the Google Earth Engine platform. This consisted of filtering Sentinel-2 Level 2A scenes (10 m spatial resolution) based on the area of interest, the sampling period, and a threshold for minimal cloud cover. The NDVI, which assesses vegetation health [43]; the Normalized Difference Water Index (NDWI), designed to delineate and enhance the presence of open water bodies [44]; ad the Coloration Index or Saturation Index (CI), developed to identify areas of bare soil and assess their degradation [45], were calculated. Additionally, a land surface temperature (LST) layer for the area of interest was constructed using Landsat 8-OLI (Level 2) imagery, specifically from the TIR 1 band (B10) with a spatial resolution of 100 m. These layers were imported into QGIS 3.16.4, where data were extracted based on the vector point layer corresponding to the georeferenced locations of the deployed AAS.

To create high-resolution land cover maps, PlanetScope imagery with a 3 m spatial resolution was used [46]. Supervised classifications were performed using QGIS 3.16.4 with the Semi-Automatic Classification Plugin (SCP). The supervised classification method employed was the Minimum Distance algorithm, utilizing the NDVI index along with the green, blue, red, and infrared bands. Four classes were identified: Impervious, low vegetation (LowVeg), high vegetation (HgVeg) and bare soil (Soil). The resulting land cover map was clipped in a 150 m radius buffer around each AAS, and the percentage area of each land cover type per sampling site was calculated using the QGIS zonal histogram algorithm. Also, a vector layer was manually drawn based on Google Earth images to identify water bodies within the study area, and the distance from each AAS to the nearest water body was calculated (S1).

Precipitation data was considered at two time lags, 14 and 30 days prior to AAS activation, based on records from the Puerto Iguazú airport weather station [47]. Selected demographic variables were obtained from National Institute of Statistics and Censuses [39] dataset, as detailed in Table 1. Elevation was not included as a variable in the analysis, as it was not considered a significant factor in the study area.

**Table 1:** Enviromental variables.

*Variable*	*Description*	*Source*	*Unit*	*Used in*
***Climatic***				
*rainfall_30*	Precipitation accumulated 30 days before the closure of each monitoring period	SMN	mm	GLMM
*globtemp*	Average air temperature	in situ	°C	GLMM
*globhum*	Average percentage of relative humidity	in situ	%	GLMM
*globwind*	Average wind speed	in situ	m/s	GLMM
*globlight*	Average light intensity	in situ	klux	GLMM
*Min_T*	Average minimum temperature recorded during the week of monitoring	SMN	°C	GLMM
*Max_T*	Average maximum temperature recorded during the week of monitoring	SMN	°C	GLMM
***Environmental***				
*watertemp*	Water temperature	in situ	°C	GLMM
*oxy*	Dissolved oxygen in the water	in situ	OD	GLMM
*pH*	Water pH	in situ		GLMM
*sal*	Salinity/conductivity	in situ	ppt	GLMM
***Vectorial***				
*albobin*	Presence/absence of *Aedes albopictus*	in situ	–	GLMM
*aegbin*	Presence/absence of *Aedes aegypti*	in situ	–	GLMM
*aeg_day*	Number of *Ae. aegypti* specimens collected, standardized by sensor active days.	in situ	–	GLMM
***Geographical***				
*Road_type*	Road types by category: paved, dirt, cobblestone and gateway	in situ	–	GLMM
*environment*	Environment by category: urban, periurban, wild environment and disturbed wild environment	by authors	–	GLMM
*gateway*	Road type where the AAS is located: Gateway	in situ	–	PCA
*paved*	Road type where the AAS is located: Paved Road	in situ	–	PCA
*cobblestone*	Road type where the AAS is located: Cobblestone	in situ	–	PCA
*dirt*	Road type where the AAS is located: Dirt Road	in situ	–	PCA
*NDVI*	Green Normalized Difference Vegetation Index	Sentinel 2 (level 2A) + GEE	–	GLMM
*NDWI*	Green Normalized Difference Water Index	Sentinel 2 (level 2A) + GEE	–	GLMM
*LST*	Land Surface Temperature	Landsat 8-OLI (Level 2) + GEE	°C	GLMM
*NDVI_win*	Normalized Difference Vegetation Index for winter	Sentinel 2 (level 2A) + GEE	–	PCA
*NDWI_win*	Normalized Difference Water Index for winter	Sentinel 2 (level 2A) + GEE	–	PCA
*CI_win*	Coloration Index for winter	Sentinel 2 (level 2A) + GEE	–	PCA
*NDVI_sum*	Normalized Difference Vegetation Index for summer	Sentinel 2 (level 2A) + GEE	–	PCA
*NDWI_sum*	Normalized Difference Water Index for summer	Sentinel 2 (level 2A) + GEE	–	PCA
*CI_sum*	Coloration Index for summer	Sentinel 2 (level 2A) + GEE	–	PCA
*Water_m*	Distance to the nearest water body	Planet Scope 3 m + QGIS	m	GLMM
*Impervious*	Impervious class	Planet Scope 3 m + QGIS	m2	GLMM & PCA
*LowVeg*	Low vegetation class (includes herbaceous or low shrubs, typically <1.5 m height)	Planet Scope 3 m + QGIS	m2	GLMM & PCA
*HgVeg*	High vegetation class (includes tall shrubs and trees, typically >1.5 m height)	Planet Scope 3 m + QGIS	m2	GLMM & PCA
*Soil*	Bare soil class	Planet Scope 3 m + QGIS	m2	GLMM
***Demographic***				
*totalpop*	Total population	INDEC 2010	inhab	GLMM & PCA
*unoccupied_rate*	Unoccupied rate	INDEC 2010	–	GLMM & PCA
*totalmale*	Total male population	INDEC 2010	inhab	PCA
*totalfemale*	Total female population	INDEC 2010	inhab	PCA
*households*	Total number of households	INDEC 2010	–	PCA
*privdwellings*	Total number of private dwellings	INDEC 2010	–	PCA
*o_priv_dwell*	Total number of occupied private dwellings	INDEC 2010	–	PCA
*house*	Total number of houses	INDEC 2010	–	PCA
*rancho*	Number of dwelling types: ‘Rancho’ (houses with adobe walls, earthen floors, and tin or thatch roofs). (INDEC - SESD Glossary, 2010)	INDEC 2010	–	PCA
*casilla*	Number of dwelling types: ‘Casilla’ (small houses built with low-quality or scrap materials). (INDEC - SESD Glossary, 2010)	INDEC 2010	–	PCA
*appartment*	Dwelling type: Apartment	INDEC 2010	–	PCA
*prem_not_built_dwell*	Dwelling type: Premises not built for dwelling	INDEC 2010	–	PCA
*room_tenants*	Dwelling type: Room with tenant	INDEC 2010	–	PCA
*motorhome*	Dwelling type: Motorhome	INDEC 2010	–	PCA
***Time***				
*days*	Ovitrap exposure, i.e., number of days that the AAS was active	Time	Date	GLMM
*month*	Monitoring months	Time	Date	GLMM

**a.** Environmental variables used to characterize the sites where the adult activity sensors (AAS) were placed, both for the assessment of site classification by principal component analysis (PCA) and for the generalized linear mixed modeling (GLMM) of *Ae. albopictus* occurrence in Puerto Iguazú, Argentina. For climatic variables measured in situ, averages were calculated from values recorded at the beginning and end of sensor activity. For variables obtained from INDEC, values were taken from the calculations for census tracts (which are geo-statistical units defined by the organization for the national census).

#### Microhabitat.

Upon activation of each AAS, the structural characteristics of the placement site (asphalt, cobblestone, dirt, or walkway) were documented, and the following parameters were recorded using a thermohygrometer: air temperature (°C), relative humidity, wind speed, and light intensity. At the end of the active period, the previous measures were repeated along with physicochemical properties of the water, including pH, dissolved oxygen (DO), salinity, and water temperature (Table 1).

### Statistical analyses

All analyses were performed using the free software platform R [48].

To evaluate the classification of the three environmental types conducted prior to sampling, a Principal Component Analysis (PCA) was performed using the package stats [48]. In this analysis, “sites” (rows) corresponded to each site where the AAS were placed, while “variables” (columns) included environmental factors such as demographic data, infrastructure details (e.g., road types), land cover and land use types, and proximity to water bodies, as outlined in Table 1.

The infestation status of *Ae. albopictus* and *Ae. aegypti,* both overall, monthly and per environmental type, was assessed by the Positivity Ovitrap Index (POI, Formula 1), commonly used in epidemiology to gauge the risk of arboviral transmission [49]. Three POI ranges were defined as indicators of low (POI ≤ 40%), moderate (41–60%) and high (>60%) entomological risk.


$$\begin{array}{c} 𝐏𝐎𝐈=\text{100}*(\text{Number of AAS with presence of }i/\text{Number of active AAS}) \end{array}$$

With *i* being either *Ae. albopictus* or *Ae. aegypti.*

Presence: at least 1 egg or 1 larva.

To assess the co-occurrence of *Ae. albopictus* and *Ae. aegypti* per environment, the significance of their association was evaluated using an adapted version of the Bayesian Proportion Difference Test proposed by Risso & Risso (2017) [50]. The null hypothesis (H0) was defined as Co-Occurrences (CO, indicating that *Ae. aegypti* and *Ae. albopictus* are both present), Exclusive Occurrence (EO, indicating that *Ae. albopictus* is present given that *Ae. aegypti* is absent) and implies no association between species, while the alternative hypothesis (H1) was defined as CO > EO, indicating association. Subsequently, for environments presenting association (p < 0.05) a diagnostic validation test was conducted using the Odds method. This methodology assesses the presence or absence of an “event” (herein, the occurrence of *Ae. albopictus*) based on a dichotomous “effect” (the occurrence of *Ae. aegypti*) and was performed at the monitoring event scale (AAS x month). The posterior odds were calculated, indicating the likelihood of *Ae. albopictus* being present in the absence/presence of *Ae. aegypti*. The Probability of Co-Occurrence (PCO), which in this context is the probability of both species being present, was then compared with the EO to ascertain the direction of the association. If PCO > EO, it indicates co-existence; otherwise, it indicates avoidance.

### Generalized linear mixed models

The occurrence of *Ae. albopictus* was analyzed using generalized linear mixed models (GLMM) with a binomial error distribution using the MASS package [51]. Initially, a pairwise correlation analysis was performed among all explanatory variables considered for inclusion in the model, using a cutoff of 𝜌 = 0.6. Subsequently, univariate models were run to assess the strength of the association between each explanatory variable and the response variable. The model was built using stepwise selection, with explanatory variables (if continuous, previously standardized) introduced one by one, along with their interactions and squared terms for continuous variables. The fixed effects evaluated included the NDVI, land cover classifications for each AAS, distance to water bodies, presence and abundance of *Ae. aegypti* (standardized by the number of days that the AAS was active), the rate of unemployment and total human population, precipitation, and environmental variables recorded *in situ* (Table 1). Random effects considered were each AAS (labelled ID), the sampling quadrants, and the four seasons. Model selection was based on the Akaike Information Criterion (AIC), with a delta AIC > 2 considered significant [52]. To assess multicollinearity among explanatory variables, the variance inflation factor (VIF) was calculated at each modeling step, and variables were only retained in the model if their VIF values were below 5 [53]. The residuals of the selected models were visually inspected to check that they follow model assumptions. Concordance was evaluated using Cohen’s Kappa estimators [54]. To measure the proportion of variability explained by fixed and random factors, conditional and marginal R^2^ values were calculated, representing the variance explained by fixed effects and by the full model (fixed + random), respectively, using the MuMIn package [55]. Finally, a spatial interpolation was performed through a spatial autocorrelation analysis using the POI values by ID and season. Since sampling points located in the INP were far apart from the urban nucleus, autocorrelation was assessed separately in INP points and non-INP points by computing Moran’s I correlograms. If significant autocorrelation was detected, a theoretical semivariogram was fitted and subsequently used to perform interpolation through the Kriging method, implemented with the gstat package [56]. If no spatial autocorrelation was found, interpolation was carried out using the Inverse Distance Weighting (IDW) method, also available in gstat.

## Results

### Environmental types: Validation and reclassification

**Fig 2:**
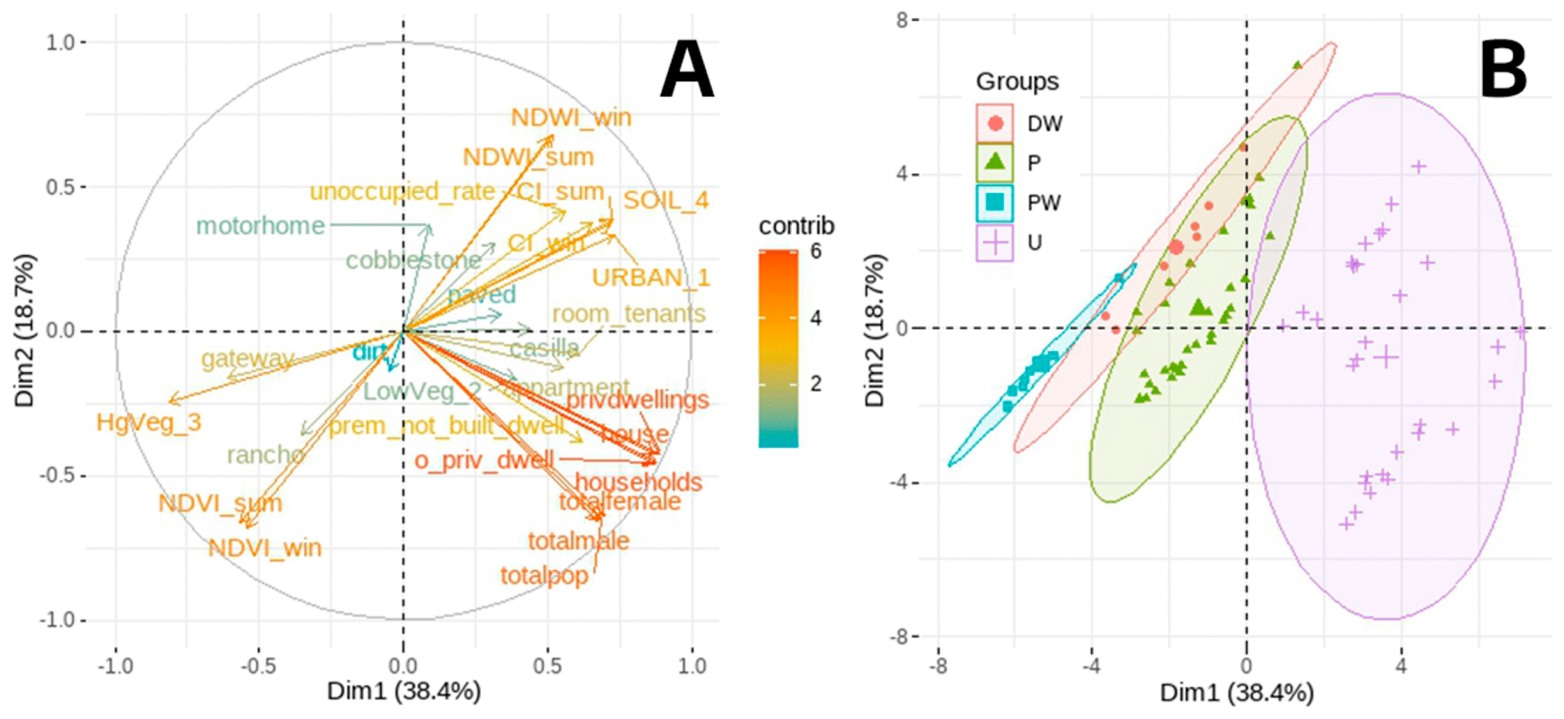
Biplot of the principal component analysis displaying the ordination of AAS deployment sites (B) as a function of environmental variables (A). **Legend:** The direction and length of the vectors indicate the magnitude of each variable’s contribution to the first two principal components, PC1 and PC2. The percentage of the total variance explained by each PC is indicated next to each axis. In **(B)**, points indicate AAS deployment sites classified by environmental type: disturbed wild (DW), periurban **(P)**, pristine wild (PW) and urban **(U)**.

The cumulative variance percentage of the first three principal components (PC) of the PCA was 70.1% (PC1 = 38.4%, PC2 = 18.7%, PC3 = 13.0%). The sites where the AAS were deployed were clustered into four main groups, based on the first two PCs. The group hereafter named “urban” included 26 out of the 35 sites previously classified as U and four sites previously classified as P (Fig 2). The cluster was defined by demographic variables such as housing types, unemployment rate, number of households and residences, and population density, showing high correlation among them as well as land cover classification predominantly impervious and bare soil, with high NDWI values and, to a lesser extent, types of paved and asphalt roads. Sites hereafter named “periurban” were 26 previously classified as P and eight previously U sites (Fig 1). These 34 sites were grouped based on intermediate vegetation cover, lower population density, higher unemployment rate, and more precarious housing types, with less emphasis on unpaved and cobblestone roads. The *a priori* defined W category included six sites resembling P areas, located at the entrance of the INP along the main paved road and near the hotel (Fig 1). These sites, characterized by high pedestrian traffic and people concentration, are hereafter referred to as “disturbed wild”, along with one site previously identified as U located on the border of Güira Oga and MNCR (Fig 1). The subset of the remaining 10 W sites, hereafter “pristine wild”, were characterized by the presence of high vegetation, high NDVI values, and footpath roads.

### Entomological sampling

**Fig 3:**
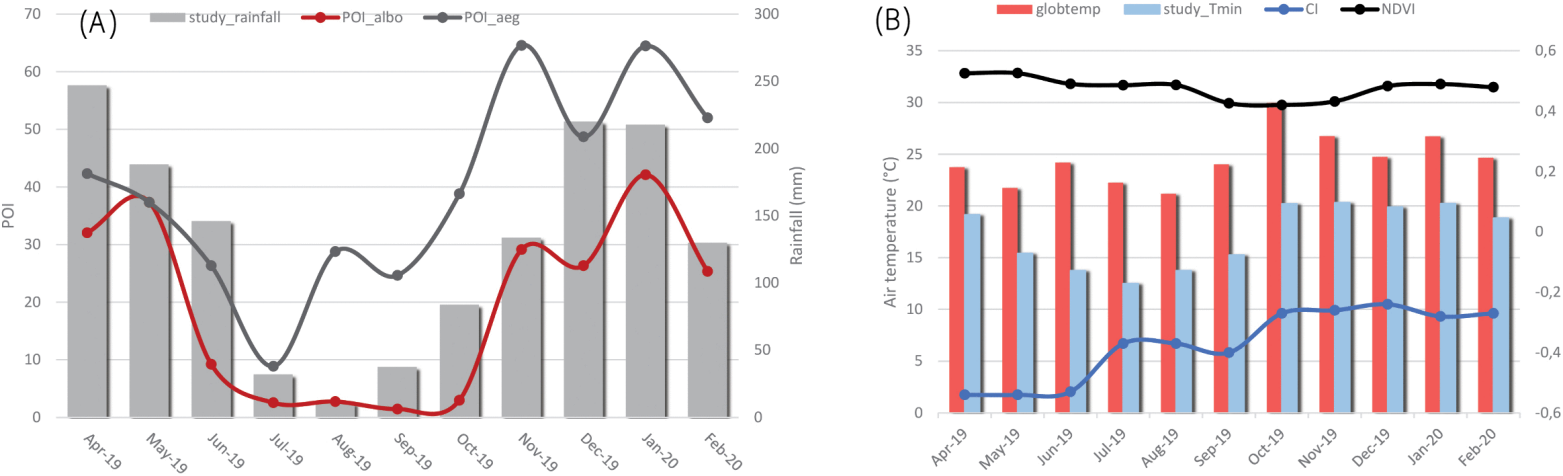
Monthly Positivity Ovitrap Index and Monthly variation spectral indices. **Legend: (A)** Monthly Positivity Ovitrap Index (POI) for *Ae. albopictus* and *Ae. aegypti*, along with monthly accumulated precipitation in bars. **(B)** Monthly variation of the spectral indices NDVI and CI; average air temperature (globtemp) and average minimal temperature (Min_T) in bars.

A total of 891 monitoring events (AAS x month) were performed, with 823 (92.5%) operating successfully, while the remaining 68 were either missing or had dried out. Of the successfully operating AAS x month, 19.6% (161) were positive for *Ae. albopictus*, and 39.9% (328) were positive for *Ae. aegypti*. In total, 1,614 *Ae. albopictus* specimens and 4,358 *Ae. aegypti* specimens were identified. The POI curve for *Ae. albopictus* exhibited a pronounced bimodal pattern, with the highest peak in January 2020 and a smaller peak in May 2019, while the lowest values were observed between June and October 2019, closely tracking precipitation patterns. For its part, the POI for *Ae. aegypti* dropped in autumn when monthly accumulated precipitation decreased but exhibited an oscillatory pattern during the winter dry season, then peaked in November 2019 and maintained a high plateau throughout the summer 2020 (Fig 3A). As for temperature, lowest positive values for both POI coincide with low winter temperatures, especially Min_T. Some minor fluctuations were also detected in the NDVI and CI seasonal patterns (Fig 3B).

**Fig 4:**
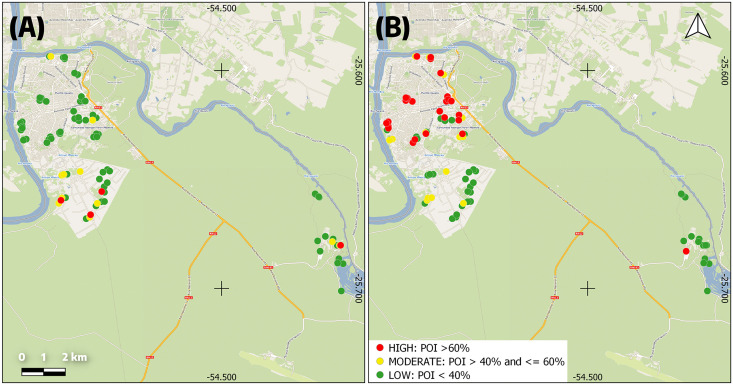
Location of the adult activity sensors (AAS). **Legend:** classified in three risk categories by their positivity ovitrap index (POI) values during the period April 2019 - February 2020 in Puerto Iguazú, Argentina, for *Ae. albopictus* (A) and *Ae. aegypti* **(B)**. Administrative boundaries obtained from the Instituto Geográfico Nacional (IGN), Argentina (https://www.ign.gob.ar/sig). This Figure contains information from OpenStreetMap and OpenStreetMap Foundation, which is made available under the Open Database License.

Regarding spatial heterogeneity, the POI for *Ae. albopictus* exhibited the highest values in periurban environments, particularly in the southern regions near the boundary of the Puerto Península Provincial Park (PPPP in Fig 1) and showed moderate to low values in both urban and wild areas (Fig 4A). Conversely, *Ae. aegypti* showed elevated values predominantly in urban environments, with a relatively uniform distribution, and moderate to low values in periurban areas. Notably, only one AAS recorded high values in the wild environment, located in a high pedestrian traffic zone (Fig 4B).

### *Aedes* dynamics of environmental type

**Fig 5:**
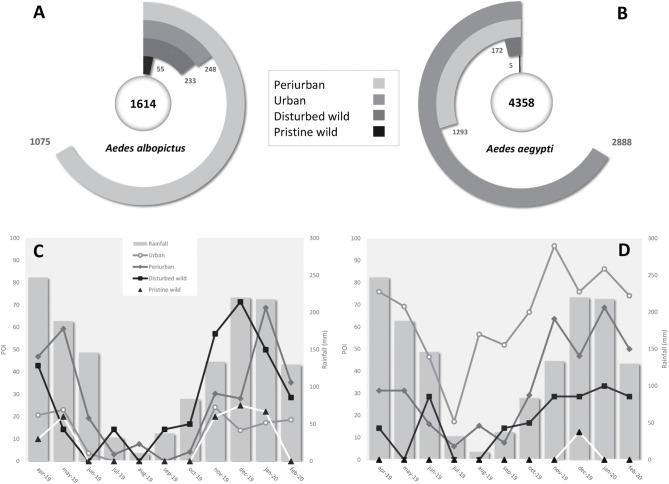
Total number of specimens of *Aedes albopictus.* **Legend:** (A) and *Ae. aegypti* (B) collected per environmental type, along with Positivity Ovitrap Index (POI) per month and environmental type overimposed on monthly cumulative precipitation (gray bars) for *Ae. albopictus* (C) and *Ae. aegypti* **(D)**.

Two-thirds of the specimens of *Ae. albopictus* identified were collected in the periurban environment, followed by urban and disturbed wild at similar percentages (14–15%, Fig 5A). The mean total abundance of *Ae. albopictus* per AAS was similar in disturbed wild (35.4) and periurban (31.7), and considerably lower in urban (7.8) and pristine wild (5.5). Highest POI values shifted from periurban in April-June to disturbed wild in July-December, and back to periurban in January-February (Fig 5C).

In contrast, the majority of *Ae. aegypti* specimens identified were collected in urban sites (Fig 5B), with highest mean abundance per AAS (96.3) and highest POI values throughout all seasons, followed by periurban particularly during spring and summer (Fig 5D) with a total mean abundance of 38.0. Values in disturbed and pristine wild environments (24.6 and 0.5, respectively) were lower compared to *Ae. albopictus*.

### Co-occurrence

The H0 was rejected for all environmental types except pristine wild, indicating an association between the presence of *Ae. albopictus* and *Ae. aegypti* in urban, periurban and disturbed wild environments. However, a significant association indicating coexistence (or avoidance), was only obtained in the urban (p < 0.001) and periurban (p = 0.027) environments. In both environmental types, the PCO was higher than the EO of *Ae. albopictus* (16% and 1% respectively in urban environments, 36% and 25% in periurban environments), indicating that the association between them is positive.

### Multivariate modeling of *Ae. albopictus* distribution

The correlation analysis among the explanatory variables (S2) showed that land cover classification variables, such as the extent of bare soil and high vegetation, exhibited high correlation values (ρ > 0.80). The latter (HgVeg) was chosen, as it better characterizes areas with minimal human intervention. Variables related to urbanization and the unemployment rate were also highly correlated, with impervious being selected due to its calculation from images closer to the sampling dates. Cumulative precipitation over 2 weeks and 30 days prior to the deactivation of the AAS were highly correlated, with the latter (rainfall_30) being selected. Between the average maximum and minimum temperature during the activation week of the AAS (Max_T and Min_T), the latter was retained, as it represents a more critical factor for larval development [57]. Finally, water and air temperatures showed ρ > 0.7; air temperature was chosen due to having more measurements (both during the activation and deactivation of the AAS).

Two alternative random structures were selected, both incorporating the season (N = 4) along with: (A) quadrants (N = 22); or (B) ID of the AAS (N = 81). In the univariate analyses, the two main variables are positively associated with the occurrence of *Ae. albopictus* were rainfall_30, the number of days that the AAS remained active (days) and Min_T. For the random structure (A), the categorical variable for road classification (Road_type) and the presence of *Ae. aegypti* (aegbin) were the most strongly associated, followed by urban land use class in a 150 m buffer around each AAS (Impervious), Land Surface Temperature (LST), air humidity (globhum), and environment (environment) to a lesser extent. For the random structure (B), along with rainfall_30 and days, environment and Impervious were equally significant, followed by LST, Road_type, distance to the nearest water body (Water_m), globhum, and the percentage of low vegetation in a 150 m buffer around each AAS (LowVeg) (see S3).

**Fig 6:**
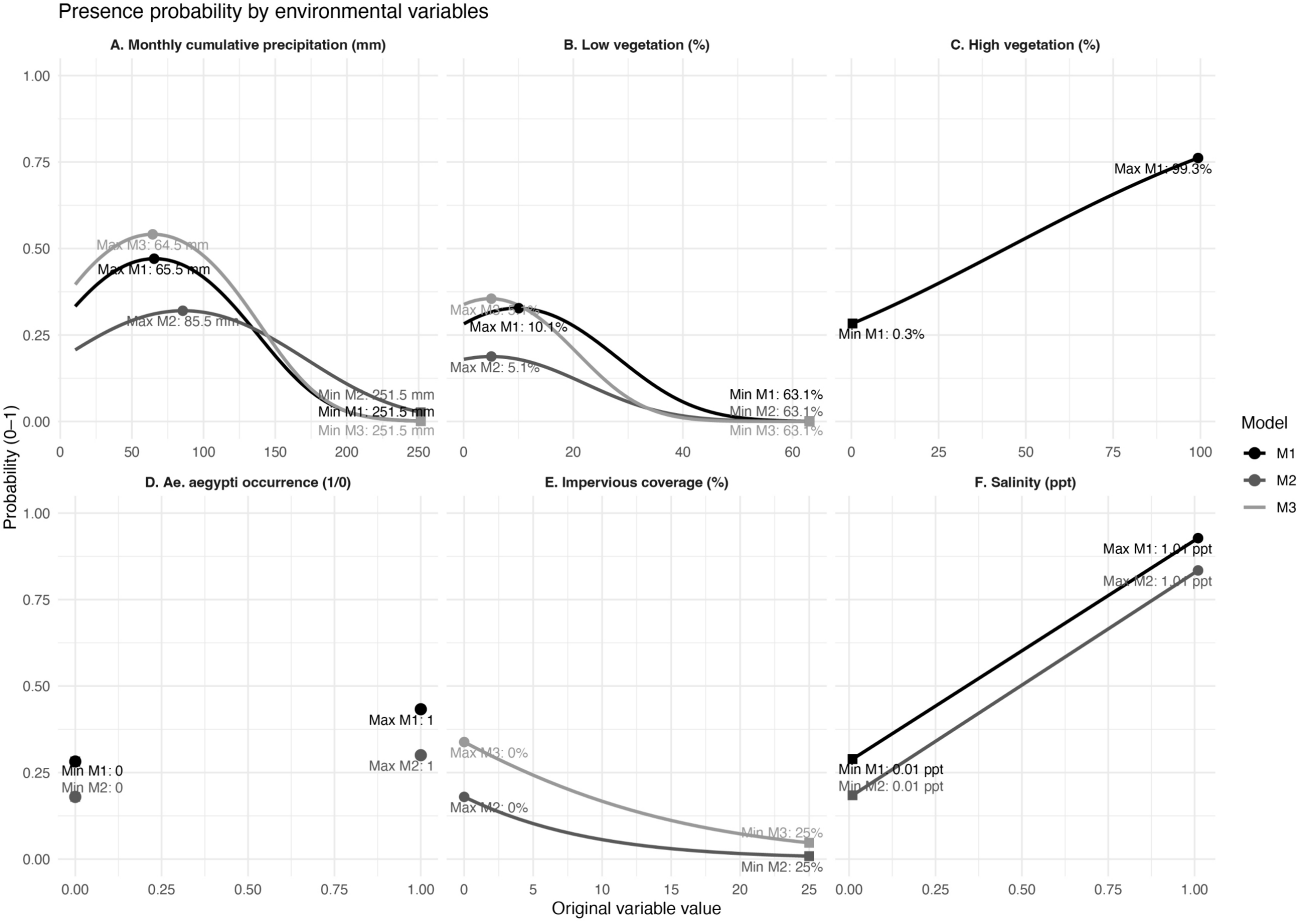
Probability of presence of *Aedes albopictus* as a function of environmental variables, according to the best-fitted generalized linear mixed models (M1, M2, and M3). **Legend:** Panels correspond to: **(A)** Monthly cumulative precipitation (mm), **(B)** Low vegetation (%), **(C)** High vegetation (%), **(D)** *Ae. aegypti* occurrence (1/0), **(E)** Impervious coverage (%), and **(F)** Salinity (ppt). Values of the explanatory variables that predict the maximum and minimum probability of occurrence of *Ae. albopictus* are indicated for each model.

Based on the specified random structures, three alternative models were attained: M1 and M2 with random structure (A), and M3 with random structure (B). The random effects accounted for 12%, 10%, and 17% of the variance in the occurrence of *Ae. albopictus*, respectively, while the fixed effects explained 47%, 50%, and 50%. The best-fitting model was M3, with a Kappa value of 0.63 (Table 2). In it, monthly precipitation exhibited a positive quadratic association that led to an increase in the probability of occurrence of *Ae. albopictus* from approximately 20% with 10.5 mm of monthly cumulative precipitation values to 54% at peak values of 64.5 mm, followed by a decline at higher precipitation values. Regarding temperature, LST showed a nonlinear inverse association, meaning that as temperature increases, the probability of presence of *Ae. albopictus* decreases, albeit with minimal influence, reaching maximum probability values (0.4%) at 16.12°C. The variable days was positively associated, indicating a higher probability of occurrence of *Ae. albopictus* as the number of ovitraps exposure days increased. LowVeg was selected as a quadratic association, with a growth area ranging from 33.8% when LowVeg is 0, to a maximum probability value of 35.5% at 5% LowVeg coverage and decreasing again to a 0% probability of occurrence of *Ae. albopictus* at coverage values around 60%. Impervious exhibited a nonlinear inverse relationship with the occurrence of *Ae. albopictus*, with maximum probability values of 34% when Impervious is 0% and gradually decreasing as impervious increases. The presence of *Ae. aegypti* was associated with the probability of occurrence of *Ae. albopictus* in the random structure models (A), taking probability values of M1 = 43.3% and M2 = 30%. Lastly, for these two models, salinity (sal) was also selected, with probabilities ranging from ≅ 23% when sal = 0.01 ppt to M1 = 93% and M2 = 83.4% when sal = 1.01 ppt in both cases (Fig 6).

**Table 2:** Log-odds coefficients (log-odds), standard errors (std error) and confidence intervals (CI).

*Predictors*	*M1* *Log-Odds*	*M1* *std Error*	*M1* *CI*	*M2* *Log-Odds*	*M2* *std Error*	*M2* *CI*	*M3* *Log-Odds*	*M3* *std Error*	*M3* *CI*
*Intercept*	-0.93	0.35	-1.62 – -0.25	-1.52	0.36	-2.22 – -0.82	-0.67	0.33	-1.32 – -0.02
*(LowVeg)* ^*2*^	-0.56	0.13	-0.82 – -0.30	-0.53	0.13	-0.78 – -0.28	-0.78	0.20	-1.17 – -0.38
*LowVeg*	0.69	0.22	0.27 – 1.12	0.54	0.16	0.23 – 0.85	0.48	0.20	-0.21 – 0.91
*rainfall_30*	1.97	0.24	1.49 – 2.44	1.41	0.21	1.00 – 1.83	2.05	0.28	1.49 – 2.60
*(rainfall_30)* ^*2*^	-1.18	0.23	-1.62 – -0.74	-0.65	0.14	-1.04 – -0.27	-1.25	0.25	-1.75 – -0.76
*days*	0.43	0.11	0.21 – 0.64	0.40	0.11	0.18 – 0.62	0.46	0.12	0.22 – 0.69
*sal*	0.27	0.10	0.07 – 0.47	0.24	0.10	0.04 – 0.46	–	–	–
*Impervious*	–	–	–	-1.03	0.22	-1.47 – -0.59	-0.74	0.26	-1.25 – -0.23
*aegbin*	0.67	0.27	-0.14 – 1.20	0.67	0.27	0.14 – 1.21	–	–	–
*LST*	-1.10	0.27	-1.63 – -0.57	–	–	–	-0.92	0.29	-1.48 – -0.35
*HgVeg*	0.58	0.27	-0.05 – 1.10	–	–	–	–	–	–
*globetemp*	–	–	–	-0.29	0.13	-0.55 – -0.04	–	–	–
	**AIC models:** 595.7 (M1), 604.2 (M2), 587.6 (M3) **Kappa:** 0.53 (M1), 0.55 (M2), 0.63 (M3) **R²m - R²c:** 47%-59% (M1), 50%-60% (M2), 50%-67% (M3)								
	**Random Effects:**								
	**τ00:** 0.98_quad (M1), 0.74_quad (M2), 1.66_ID (M3)								
	**σ00 Seasons:** 0.00 (M1), 0.08 (M2), 0.04 (M3)								
	**ICC:** 0.23 (M1), 0.20 (M2), 0.34 (M3)								
	AIC Null models: Quad = 680.6, ID = 679.2								

b. Log-Odds coefficients (Log-Odds), standard errors (std Error) and confidence intervals (CI) for the predictors included in generalized linear mixed models M1, M2, and M3 for the probability of occurrence of *Ae. albopictus* in Puerto Iguazú, Argentina, during the period April 2019 - February 2020. The abbreviations of the explanatory variables selected in these models are presented in Table 1. Model fit metrics such as AIC, Kappa, and both marginal and conditional R^2^ are included, along with random effects and the intraclass correlation coefficient (ICC).

**Fig 7:**
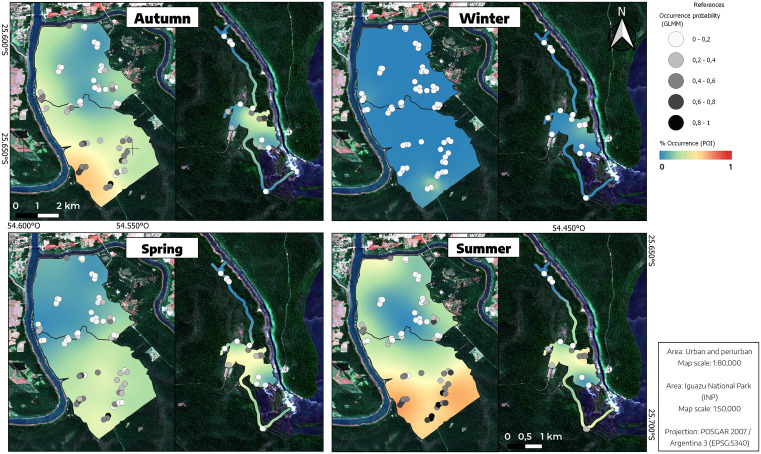
Spatial interpolations using Kriging and IDW of the probability of occurrence of *Aedes albopictus*, based on POI values and predictions from model M3. **Legend:** The points represent the locations of the AAS, with their predicted probabilities (0–1) shown in grayscale. The interpolated probability of occurrence is displayed with a color gradient ranging from blue tones (low probability, close to 0) to red tones (high probability, close to 1). Administrative boundaries obtained from the Instituto Geográfico Nacional (IGN), Argentina (https://www.ign.gob.ar/sig). This Figure include Landsat 8 Operational Land Imager (OLI) imagery courtesy of the **U.**S. Geological Survey (USGS), available in the public domain (April 2019 – February 2020).

The geostatistical projections of model M3 are shown in Fig 7. The IDW interpolation method was employed during the winter season for both the INP and non-INP groups due to the absence of significant spatial autocorrelation, as evidenced by Moran’s I values ranging from −0.05 to 0.04 (p ≥ 0.06). In autumn, Kriging was applied for the non-INP group, where short-range spatial dependence was evident (~0.9 km, Moran’s I = 0.20, p = 1.0 × 10^-6^; ~ 2.6 km, Moran’s I = 0.06, p = 0.009), while IDW was maintained for INP (p > 0.3). In the spring, Kriging was employed for both groups due to the presence of significant positive correlations at short distances (INP: ~ 1.3 km, Moran’s I = 0.10, p = 0.0025; non-INP: ~ 0.9–2.6 km, Moran’s I = 0.12–0.10, p < 0.001). During the summer season, Kriging was employed for the non-INP group, given the pronounced clustering observed (~0.9 km, I = 0.27, p = 1.1 × 10^-10^). Conversely, IDW was maintained for INP, as the calculation of a correlogram was precluded by data limitations. Autumn and spring exhibit similar probabilities of occurrence, although the trend suggests that the species remains more prevalent after summer, whereas its distribution after winter becomes spatially more restricted. At the spatial level, a clear zoning pattern is observed, with a major hotspot located in the peri-urban environment near the PPPP and PHDNR reserves (see Fig 1). This area is characterized by lower housing density and infrastructure, while patches of wild vegetation are preserved and intermixed with intensive subsistence agriculture. During periods of higher probabilities, the borders of the urban area “light up”, but the species is virtually absent in the central urban core, where urbanization is more consolidated. The disturbed wild environment, particularly the public-use sector of INP, shows a localized hotspot in autumn that persists slightly into winter and re-emerges in spring, spreading more evenly and continuing into summer. In contrast, in the pristine wild environment, almost no probability of occurrence is observed throughout the seasons, except for a slight increase in the southern part of the area closest to the circuits with the highest human traffic in the INP during summer.

## Discussion

Findings in sub-tropical Argentina provide robust evidence for a spatially stratified distribution of *Ae. albopictus*, with the periurban environment acting as the most favorable setting for its proliferation. This pattern aligns with previous work documenting increased vector presence in ecotones and buffer zones [26,58]. Transitional environments, where anthropogenic landscapes interlace with remnants of native forest, create pronounced edge effects that appear to facilitate vector presence—an observation also documented for *Haemagogus* spp., a sylvatic vector of yellow fever, in similar contexts [59].

Consistent with the observations of Lazaric et al. [60], the area labelled herein as disturbed wild, located within public use zones of the INP, is associated with features typical of periurban environments and also harbored *Ae. albopictus* throughout the year. In this context, vector presence appears to be associated with anthropogenic interventions that lead to land-use changes affecting natural environments. In contrast, the spatial stratification observed for *Ae. aegypti* demonstrates a clear dominance of the species in highly urbanized settings, in coincidence with the literature [19] and supported by the co-occurrence results presented here, which revealed a positive association between both species, with *Ae. aegypti* dominating in urban environments. Regarding temporal distribution, our study revealed year-round *Ae. aegypti* oviposition in both urban and periurban environments, which may be an indicator of dengue endemicity. However, the establishment of endemic dengue also depends on the concurrence of additional factors, such as persistent virus circulation, adequate susceptible host density, and suitable climatic conditions [61]. This finding underscores the need to intensify studies in other localities to compare and validate these results.

Building on Espinosa et al. [58], we observed that, as urbanization expanded and intensified over space and time within the study area, *Ae. albopictus* progressively shifted toward more recently urbanized zones with lower levels of imperviousness. Consistent with Reiskind et al. [62], the species exhibited greater stability at the edges of densely vegetated areas. Therefore, land-use changes can be considered a risk factor, and their identification and monitoring should be regarded as an early warning indicator of potential colonization by *Ae. albopictus*. This highlights the critical role of natural environments in maintaining ecosystem functions with direct implications for public health, as land-use change and habitat fragmentation have been identified as contributing factors in the emergence and transmission of zoonotic diseases [30,63].

When analyzing our results in conjunction with previous studies in the region, we observe heterogeneity in vector distribution as a function of urbanization. Lizuain et al. [13] assessed the occurrence of *Ae. albopictus* in two localities with contrasting infrastructure and urban development: Eldorado (26°24′00″S; 54°38′00″W), a large city with high urban density, and Colonia Aurora (27°28′29″S; 54°31′28″W), a smaller town with a rural profile and greater connectivity to natural areas. In Eldorado, *Ae. albopictus* was reported with low relative abundance, whereas in Colonia Aurora, it showed the highest relative abundance of *Ae. albopictus* recorded in Argentina to date in co-dominance with *Ae. aegypti*. Accordingly, the abundance of *Ae. albopictus* across these localities, along with the areas examined in the present study, can be represented by a bell-shaped pattern: low to moderate presence in Eldorado and the urban area of Puerto Iguazú, gradually increasing in the periurban of Puerto Iguazú, peaking in Colonia Aurora, then declining in the disturbed wild areas of the INP, and reaching minimal levels in the pristine wild zones of the INP [13].

Regarding the use of AAS, our findings align with those of Fernandes Silva Chagas do Nascimento et al. (2025) [64] who stated that this methodology is an effective method for detecting the presence or absence of *Ae. aegypti* and *Ae. albopictus.* Although it does not directly assess adult mosquito populations, it effectively captures population trends and fluctuations in both species.

Seasonal dynamics of *Ae. albopictus* was primarily driven by precipitation, as previously reported [19,65], presumably due to water availability acting as a limiting factor in peridomestic environments [28], while minimum temperature thresholds had a secondary influence. At the microhabitat scale, the physicochemical properties of water within containers were also associated with *Ae. albopictus* presence. Higher salinity was positively associated, likely reflecting the accumulation of decomposing organic matter. Organic detritus releases dissolved ions—such as nitrates, phosphates, and humic substances—that enrich aquatic habitats and enhance larval development [66]. This process was especially evident in ecotonal areas, where containers are frequently surrounded by vegetation, contrasting with urban environments dominated by clean water in artificial containers. Our temporal models identified a 30‑day post‑rainfall window as the optimal period for *Ae. albopictus* detection, representing the lag between water availability and peak oviposition. This should guide intensified monitoring in high‑risk strata. Future studies should address this interaction between physicochemical and environmental factors, which may help explain why *Ae. albopictus* tends to establish more strongly in transitional areas with anthropogenic influence and surrounding vegetation, compared to densely urbanized settings or pristine forest environments.

However, it is important to point out that the sampling year was relatively atypical, with slightly higher temperatures than the historical average, although the characteristic wet–dry seasonal dynamics were preserved. Extending the study over a longer temporal scale is therefore recommended (see S4).

Upon analysis of the spatial autocorrelation results, it was evident that this phenomenon was manifested most prominently during the spring and summer months. During the autumn season, this effect was observed to a lesser extent, however, this observation pertained exclusively to the non-INP group. The winter season, conversely, exhibited an absence of any discernible autocorrelation. In the non-INP group, two significant distance classes were detected (~ 0.9 km and ~ 2.6 km), suggesting the existence of two clustering patterns, possibly associated with the combination of urban and periurban environments within the same group. These results are consistent with the seasonal patterns of probability of occurrence of *Ae. albopictus*, which peaks in summer and decreases almost completely in winter. In order to support risk stratification for the transmission of zoonotic diseases in the study area, and based mostly on remote sensing data, we defined an environmental stratification of the study area into four sectors of epidemiological interest: urban, periurban, pristine wild and disturbed wild. Periurban strata concentrate the majority of *Ae. albopictus* occurrence, following an 80/20 pattern where approximately 20% of the landscape accounts for 80% of vector presence. This is consistent with the heterogeneity principle highlighted by Woolhouse et al. (1997), who demonstrated that targeting high-transmission foci maximizes control efficiency [67]. The ability to stratify environments by identifying variables that characterize ecological or demographic areas of interest, and that show significant correlation with the spatiotemporal distribution of *Ae. albopictus*, represents a valuable monitoring tool for entomological risk stratification and for guiding zoonotic disease prevention efforts.

A strong association was found between the spatiotemporal distribution of *Ae. albopictus* and land-use changes resulting from unplanned urban development. These landscapes represent high-risk scenarios for the spread of zoonotic diseases. Colonization of the vector is postulated to be driven by a delicate balance within the landscape, wherein moderate human intervention provides access to a diversity of larval habitats (both natural and artificial), while maintaining sufficient connectivity to native forest patches or dense and heterogeneous vegetation that offer blood sources and resting sites. We propose that the colonization of new environments by *Ae. albopictus* is significantly associated with recent anthropogenic disturbance in wild areas, and that the species exhibits metapopulation dynamics—where periurban zones function as source habitats, while both anthropized and pristine areas act as sinks. For surveillance, the observed spatial autocorrelation distances (~0.9 km and ~2.6 km) support a stratified, risk‑based design. In the high‑risk strata identified above, deploying ovitraps at higher density year‑round would maximize detection efficiency. For vector control, the co‑occurrence of *Ae. albopictus* and *Ae. aegypti* in transitional landscapes requires integrated actions targeting both species.

Thus, the risks associated with zoonotic disease transmission are not only linked to socioeconomic conditions and access to basic public services, but also to local socio-environmental factors—such as the absence of political-administrative tools for environmental land-use planning—and regional variables related to climate dynamics. Specifically, embedding the identified stratification criteria into local environmental land-use planning. We hypothesize that territorial planning strategies could mitigate health risks. In line with the “urban One Health” paradigm proposed by Ellwanger et al. [68], promoting sustainable urbanization with adequate infrastructure may not only limit mosquito proliferation but also improve broader population health outcomes.

## Conclusions

In conclusion, our findings reveal that *Ae. albopictus* establishment in the subtropical triple border region is spatially structured, with highest occurrence in peri‑urban and disturbed wild areas where anthropogenic intervention is moderate but unplanned, reflecting a lack of environmental land‑use planning as mandated by environmental policy. This reflects the loss of nature’s contributions to public health, as the natural population regulation of disease vectors is affected by unplanned landscape transformation, creating new ecological niches for their proliferation. These transitional landscapes act as ecotones that concentrate the species presence, suggesting that land‑use change, rather than urbanization alone, is a key driver. From a surveillance perspective, this spatial stratification allows a shift from uniform, city‑wide sampling to a stratified, risk‑based design: efforts should concentrate on the peri‑urban buffers, public‑use zones in protected areas, and recently urbanized peripheries that accounts for the majority of vector occurrence an application of the 80/20 principle for resource allocation. In these high‑risk strata, deploying ovitraps at higher density year‑round, with intensified monitoring after rainfall events over 30 days (the optimal window identified in our models), would maximize detection efficiency. For vector control, cost‑effectiveness requires integrated actions that target both *Ae. albopictus* and co‑occurring *Ae. aegypti* using a combination of artificial and natural container management, tailored to each environmental stratum. Ultimately, embedding these criteria into local land‑use planning would transform vector control from a reactive to a preventive, intersectoral intervention aligned with the “Urban One Health”conceptual framework.

## Supporting information

S1 Fig Land cover classification. **Legend:** Four land cover classes were identified (impervious, low vegetation, high vegetation, and soil), clipped within 150 m buffers around adult activity sensor (AAS) sites; water bodies were digitized from Google Earth for distance calculations. Administrative boundaries obtained from the Instituto Geográfico Nacional (IGN), Argentina (https://www.ign.gob.ar/sig). This Figure includes Landsat 8 Operational Land Imager (OLI) imagery courtesy of the U.S. Geological Survey (USGS), available in the public domain (April 2019 – February 2020) and information from OpenStreetMap and OpenStreetMap Foundation, which is made available under the Open Database License.
(TIF)

S2 Fig Cluster dendrogram of explanatory variables. Legend: Variables selected for inclusion in the GLMMs were highlighted, based on groups with pairwise correlations greater than 0.6.
(TIF)

S1 Table Results of univariate GLMM analyses for the occurrence of *Ae. albopictus* (albobin) using two alternative random structures: right, structure A quadrants (quad) + seasons; left, structure B AAS ID+ seasons. In both cases, rainfall_30, days active, and Min_T were consistently significant predictors of *Aedes albopictus* occurrence. Additional variables with strong associations included Road_type, Impervious cover, LST, and environment, while most other predictors were non-significant. See Table 1 for variable definitions.
(DOCX)

S3 Fig Comparison of monthly rainfall and air temperature between the study period and historical climatology. **Legend:** Monthly precipitation (bars) and air temperature (lines) during the sampling period (April 2019–February 2020) compared with the historical climatological averages for the period 1992–2018. Grey bars represent observed precipitation during the study period, while yellow bars indicate historical monthly averages. Red lines correspond to maximum air temperature (Tmax) and blue lines to minimum air temperature (Tmin), with darker tones representing historical values and lighter tones representing the study period.
(TIF)

S2 Table Environmental and biotic data set used for modeling analyzed. **Legend:** Dataset used to assess the occurrence of *Aedes albopictus* through generalized linear mixed models (GLMM) and co-occurrence analyses. The dataset includes environmental, climatic, microhabitat, and demographic variables associated with each sampling unit, as well as vector presence/absence data.
(XLSX)

S3 Table Geographic coordinates of adult activity sensor (AAS) sampling sites. **Legend:** This file contains the geographic coordinates of all sampling sites where adult activity sensors (AAS, ovitraps) were deployed in Puerto Iguazú, Misiones Province, Argentina, during the study period (April 2019 – February 2020).
(XLSX)

## References

[1] Girard M, Nelson CB, Picot V, Gubler DJ. Arboviruses: A global public health threat. Vaccine. 2020;38(24):3989–94. doi: 10.1016/j.vaccine.2020.04.011 32336601 PMC7180381

[2] Reisen WK. Landscape epidemiology of vector-borne diseases. Annu Rev Entomol. 2010;55:461–83. doi: 10.1146/annurev-ento-112408-085419 19737082

[3] Emmanuel NN, Loha N, Okolo MO, Ikenna OK. Landscape epidemiology: An emerging perspective in the mapping and modelling of disease and disease risk factors. Asian Pacific Journal of Tropical Disease. 2011;1(3):247–50. doi: 10.1016/s2222-1808(11)60041-8

[4] Lambrechts L, Scott TW, Gubler DJ. Consequences of the expanding global distribution of Aedes albopictus for dengue virus transmission. PLoS Negl Trop Dis. 2010;4(5):e646. doi: 10.1371/journal.pntd.0000646 20520794 PMC2876112

[5] Alencar J, Ferreira de Mello C, Brisola Marcondes C, Érico Guimarães A, Toma HK, Queiroz Bastos A, et al. Natural Infection and Vertical Transmission of Zika Virus in Sylvatic Mosquitoes Aedes albopictus and Haemagogus leucocelaenus from Rio de Janeiro, Brazil. Trop Med Infect Dis. 2021;6(2):99. doi: 10.3390/tropicalmed6020099 34207935 PMC8293354

[6] Gratz NG. Critical review of the vector status of Aedes albopictus. Med Vet Entomol. 2004;18(3):215–27. doi: 10.1111/j.0269-283X.2004.00513.x 15347388

[7] European Centre for Disease Prevention and Control, European Food Safety Authority. Aedes albopictus – current known distribution: June 2025. ECDC Mosquito Maps. 2025. https://www.ecdc.europa.eu/en/publications-data/aedes-albopictus-current-known-distribution-june-2025/

[8] Sprenger D, Wuithiranyagool T. The discovery and distribution of Aedes albopictus in Harris County, Texas. J Am Mosq Control Assoc. 1986;2(2):217–9. 3507493

[9] Moore CG, Mitchell CJ. Aedes albopictus in the United States: ten-year presence and public health implications. Emerg Infect Dis. 1997;3(3):329–34. doi: 10.3201/eid0303.970309 9284377 PMC2627635

[10] Forattini OP. Identificação de *Aedes (Stegomyia) albopictus* (Skuse) no Brasil. Rev Saude Publica. 1986;20:244–5. 3809982 10.1590/s0034-89101986000300009

[11] La Corte dos Santos R. Updating of the distribution of Aedes albopictus in Brazil (1997-2002). Rev Saude Publica. 2003;37(5):671–3. 14569346

[12] Rossi GC, Pascual NT, Krsticevic FJ. First record of Aedes albopictus (Skuse) from Argentina. J Am Mosq Control Assoc. 1999;15(3):422. 10480134

[13] Lizuain AA, Maffey L, Leporace M, Garzón M, Schweigmann N, Santini MS. Factors associated with the presence and abundance of Aedes albopictus and Aedes aegypti (Diptera: Culicidae): Perspectives from larval habitat-scale and neighbourhood-scale analyses in the Argentine subtropics. Med Vet Entomol. 2025;39(2):361–72. doi: 10.1111/mve.12785 39739316

[14] Lizuain AA, Muttis E, Leporace M, Cano ME, Acardi S, Sánchez Gavier F, et al. Mosquito vectors of yellow fever virus in areas of epidemiological risk in northeastern Argentina. Ecol Austral. 2024;:393–400. doi: 10.25260/ea.24.34.2.0.2254

[15] Martín ME, Alonso AC, Faraone J, Stein M, Estallo EL. Aedes aegypti and Aedes albopictus abundance, landscape coverage and spectral indices effects in a subtropical city of Argentina. bioRxiv. 2022. doi: 10.1101/2022.01.11.475665

[16] World Health Organization, Convention on Biological Diversity. Connecting global priorities: biodiversity and human health: a state of knowledge review. WHO/CBD. 2015. doi: 10.13140/RG.2.1.3679.6565

[17] Patz JA, Daszak P, Tabor GM, Aguirre AA, Pearl M, Epstein J, et al. Unhealthy landscapes: Policy recommendations on land use change and infectious disease emergence. Environ Health Perspect. 2004;112(10):1092–8. doi: 10.1289/ehp.6877 15238283 PMC1247383

[18] de Castro MC, Monte-Mór RL, Sawyer DO, Singer BH. Malaria risk on the Amazon frontier. Proc Natl Acad Sci U S A. 2006;103(7):2452–7. doi: 10.1073/pnas.0510576103 16461902 PMC1413719

[19] Sallam MF, Fizer C, Pilant AN, Whung P-Y. Systematic Review: Land Cover, Meteorological, and Socioeconomic Determinants of Aedes Mosquito Habitat for Risk Mapping. Int J Environ Res Public Health. 2017;14(10):1230. doi: 10.3390/ijerph14101230 29035317 PMC5664731

[20] Evans MV, Hintz CW, Jones L, Shiau J, Solano N, Drake JM, et al. Microclimate and Larval Habitat Density Predict Adult Aedes albopictus Abundance in Urban Areas. Am J Trop Med Hyg. 2019;101(2):362–70. doi: 10.4269/ajtmh.19-0220 31190685 PMC6685558

[21] Garzón MJ, Maffey L, Lizuain A, Soto D, Diaz PC, Leporace M, et al. Temperature and photoperiod effects on dormancy status and life cycle parameters in Aedes albopictus and Aedes aegypti from subtropical Argentina. Med Vet Entomol. 2021;35(1):97–105. doi: 10.1111/mve.12474 32827166

[22] Little E, Biehler D, Leisnham PT, Jordan R, Wilson S, LaDeau SL. Socio-Ecological Mechanisms Supporting High Densities of Aedes albopictus (Diptera: Culicidae) in Baltimore, MD. J Med Entomol. 2017;54(5):1183–92. doi: 10.1093/jme/tjx103 28605549 PMC5850657

[23] Segura NA, Muñoz AL, Losada-Barragán M, Torres O, Rodríguez AK, Rangel H, et al. Minireview: Epidemiological impact of arboviral diseases in Latin American countries, arbovirus-vector interactions and control strategies. Pathog Dis. 2021;79(7):ftab043. doi: 10.1093/femspd/ftab043 34410378

[24] Rubio A, Cardo MV, Vezzani D, Carbajo AE. Aedes aegypti spreading in South America: new coldest and southernmost records. Mem Inst Oswaldo Cruz. 2020;115:e190496. doi: 10.1590/0074-02760190496 32401999 PMC7207151

[25] Lounibos LP, Kramer LD. Invasiveness of Aedes aegypti and Aedes albopictus and Vectorial Capacity for Chikungunya Virus. J Infect Dis. 2016;214(suppl 5):S453–8. doi: 10.1093/infdis/jiw285 27920173 PMC5137242

[26] Braks MAH, Honório NA, Lourençqo-De-Oliveira R, Juliano SA, Lounibos LP. Convergent habitat segregation of Aedes aegypti and Aedes albopictus (Diptera: Culicidae) in southeastern Brazil and Florida. J Med Entomol. 2003;40(6):785–94. doi: 10.1603/0022-2585-40.6.785 14765654

[27] Rey JR, Lounibos LP. Ecología de Aedes aegypti y Aedes albopictus en América y transmisión de enfermedades. Biomédica. 2015;35:177–85. 10.7705/biomedica.v35i2.2514 26535539

[28] Cândido EL, da Silva UM, de Souza Costa AR, de Oliveira Morais EES, da Silva Leite GM, Oliveira CW. Aedes (Stegomyia) albopictus in rural areas in Brazil: first record in the state of Ceará. Glob J Ecol. 2023;8: 97–102. doi: 10.17352/gje.000088

[29] Administración deParques Nacionales. Plan de gestión del Parque Nacional Iguazú: período 2017–2023. Puerto Iguazú: APN; 2017.

[30] Ellwanger JH, Fearnside PM, Ziliotto M, Valverde-Villegas JM, Veiga ABGD, Vieira GF, et al. Synthesizing the connections between environmental disturbances and zoonotic spillover. An Acad Bras Cienc. 2022;94(suppl 3):e20211530. doi: 10.1590/0001-3765202220211530 36169531

[31] Gardner L, Sarkar S. A global airport-based risk model for the spread of dengue infection via the air transport network. PLoS One. 2013;8(8):e72129. doi: 10.1371/journal.pone.0072129 24009672 PMC3756962

[32] Ministerio deSalud de la Nación. Boletín Epidemiológico Nacional. Argentina. 2024. https://www.argentina.gob.ar/salud/boletin-epidemiologico-nacional

[33] Pan American Health Organization, World Health Organization. Epidemiological alert: increase in chikungunya in the Region of the Americas. PAHO/WHO. 2023. https://www.paho.org/es/alertas-epidemiologicas

[34] Vezzani D, Carbajo AE. Aedes aegypti, Aedes albopictus, and dengue in Argentina: current knowledge and future directions. Mem Inst Oswaldo Cruz. 2008;103(1):66–74. doi: 10.1590/s0074-02762008005000003 18327504

[35] Pereira Dos Santos T, Roiz D, Santos de Abreu FV, Luz SLB, Santalucia M, Jiolle D, et al. Potential of Aedes albopictus as a bridge vector for enzootic pathogens at the urban-forest interface in Brazil. Emerg Microbes Infect. 2018;7(1):191. doi: 10.1038/s41426-018-0194-y 30482898 PMC6258732

[36] Servicio Meteorológico Nacional. Atlas climático. Ministerio de Defensa: Argentina. 2024. https://www.smn.gob.ar/clima/atlasclimatico/

[37] Berrozpe PE, Lamattina D, Santini MS, Araujo AV, Torrusio SE, Salomón OD. Spatiotemporal dynamics of Lutzomyia longipalpis and macro‐habitat characterization using satellite images in a leishmaniasis‐endemic city in Argentina. Medical Vet Entomology. 2018;33(1):89–98. doi: 10.1111/mve.12334 30198066

[38] Furlan V, Pochettino ML, Hilgert NI. Management of Fruit Species in Urban Home Gardens of Argentina Atlantic Forest as an Influence for Landscape Domestication. Front Plant Sci. 2017;8:1690. doi: 10.3389/fpls.2017.01690 29033964 PMC5625568

[39] Instituto Nacional de Estadística y Censos. Censo nacional de población, hogares y vivienda. Buenos Aires, Argentina. 2010.

[40] Silver JB. Mosquito ecology: field sampling methods. Dordrecht: Springer Netherlands. 2008.

[41] Dias R, de Mello CF, Santos GS, Carbajal-de-la-Fuente AL, Alencar J. Vertical Distribution of Oviposition and Temporal Segregation of Arbovirus Vector Mosquitoes (Diptera: Culicidae) in a Fragment of the Atlantic Forest, State of Rio de Janeiro, Brazil. Trop Med Infect Dis. 2023;8(5):256. doi: 10.3390/tropicalmed8050256 37235304 PMC10221014

[42] Forattini OP. Culicidologia médica: identificação, biologia e epidemiologia. São Paulo: EDUSP. 2002.

[43] Shit PK, Pourghasemi HR, Adhikary PP, Bhunia GS, Sati VP. Forest resources resilience and conflicts. Amsterdam: Elsevier. 2021.

[44] Fatima N, Alamgir A, Khan MA, Fatima SU, Malik E. Monitoring of environmental resources using NDVI and NDWI in the coastal areas of Sindh, Pakistan. Environ Monit Assess. 2022;194:561.35789439

[45] Escadafal R, Belghit A, Ben-Moussa A. Indices spectraux pour la télédétection de la dégradation des milieux naturels en Tunisie aride. In: Proceedings of the 6th International Symposium on Physical Measurements and Signatures in Remote Sensing; 1994. 17–21.

[46] Planet Labs Inc. Education and research programme: satellite imagery. Planet Explorer. 2019–20. https://www.planet.com/

[47] Servicio Meteorológico Nacional. Datos meteorológicos de la estación Iguazú Aero (2019–2020). Ministerio de Defensa: Argentina. 2021. https://www.smn.gob.ar

[48] RCore Team. R: a language and environment for statistical computing. Version 4.5.1. Vienna: R Foundation for Statistical Computing. 2025. https://www.R-project.org/

[49] Pereira LP, Teixeira CW, Rodrigues MFR, Marteleto NC, da Silva SIA, Pujoni DGF, et al. Contribution of ovitrap in the control of the Aedes aegypti vector and reduction of dengue cases in the municipality of Ibirité in Minas Gerais. UNINGÁ Rev. 2022;37:eURJ4418. doi: 10.46311/2178-2571.37.eurj4418

[50] Risso MA, Risso P. Introducción a la estadística bayesiana: uso de lenguaje R y WinBUGS. La Plata: Vuelta a Casa. 2017.

[51] Venables WN, Ripley BD. Modern applied statistics with S. 4th ed. New York: Springer. 2002.

[52] Zuur AF, Ieno EN, Smith GM. Analysing ecological data. New York: Springer. 2007.

[53] Zuur AF, Ieno EN, Walker NJ, Saveliev AA, Smith GM. Mixed effects models and extensions in ecology with R. New York: Springer. 2009. doi: 10.1007/978-0-387-87458-6

[54] Cohen J. A coefficient of agreement for nominal scales. Educ Psychol Meas. 1960;20:37–46. doi: 10.1177/001316446002000104

[55] Barton K. MuMIn: multi-model inference. R package version 1.15.6. 2016. https://CRAN.R-project.org/package=MuMIn

[56] Pebesma EJ. Multivariable geostatistics in S: the gstat package. Comput Geosci. 2004;30:683–91. doi: 10.1016/j.cageo.2004.03.012ALTO

[57] Alto BW, Juliano SA. Precipitation and temperature effects on populations of Aedes albopictus (Diptera: Culicidae): implications for range expansion. J Med Entomol. 2001;38(5):646–56. doi: 10.1603/0022-2585-38.5.646 11580037 PMC2579929

[58] Espinosa M, Weinberg D, Gómez A, Abril M. Primer registro de Aedes albopictus (Skuse) (Diptera: Culicidae) en la Ciudad de Puerto Iguazú, Misiones, Argentina. Rev Argent Zoonosis Enferm Infecc Emerg. 2012;7:24–6. https://mundosano.org/wp-content/uploads/2018/03/primer-registro.pdf

[59] Siches JA, Berrozpe PE, Rossi GC, Salomón OD, García JJ. Haemagogus leucocelaenus (Diptera: Culicidae), the potential wild vector of yellow fever in the border zone of northern Misiones, Argentina. Rev Soc Entomol Argent. 2021;80:136–41. doi: 10.25085/rsea.800410

[60] Lazaric ML, Fernández MS, Lestani EA, Pérez AA. Actividad de oviposición de Aedes aegypti y Aedes albopictus en tres ambientes con distintos grados de antropización en el área de uso público del Parque Nacional Iguazú, Misiones, Argentina. In: Libro de resúmenes de la X Jornada Regional de Mosquitos. Mar del Plata: INBIOTEC-CONICET & FIBA; 2016. 81. https://fibamdp.wordpress.com/wp-content/uploads/2016/03/xjrm-libro-resc3bamenes.pdf

[61] Nakase T, Giovanetti M, Obolski U, Lourenço J. Population at risk of dengue virus transmission has increased due to coupled climate factors and population growth. Commun Earth Environ. 2024;5(1). doi: 10.1038/s43247-024-01639-6

[62] Reiskind MH, Griffin RH, Janairo MS, Hopperstad KA. Mosquitoes of field and forest: the scale of habitat segregation in a diverse mosquito assemblage. Med Vet Entomol. 2017;31(1):44–54. doi: 10.1111/mve.12193 27759165

[63] Intergovernmental Science-Policy Platform on Biodiversity and Ecosystem Services. The IPBES assessment report on land degradation and restoration. Montanarella L, Scholes R, Brainich A. Bonn: IPBES Secretariat. 2018.

[64] Fernandes Silva Chagas do Nascimento R, da Silva Xavier A, Ayllón Santiago T, Câmara DCP, Dos Reis IC, Delatorre E, et al. Systematic Review of the Ovitrap Surveillance of Aedes Mosquitoes in Brazil (2012-2022). Trop Med Infect Dis. 2025;10(8):212. doi: 10.3390/tropicalmed10080212 40864115 PMC12390002

[65] Kache PA, Eastwood G, Collins-Palmer K, Katz M, Falco RC, Bajwa WI, et al. Environmental Determinants of Aedes albopictus Abundance at a Northern Limit of Its Range in the United States. Am J Trop Med Hyg. 2020;102(2):436–47. doi: 10.4269/ajtmh.19-0244 31833467 PMC7008348

[66] Westby KM, Adalsteinsson SA, Biro EG, Beckermann AJ, Medley KA. Aedes albopictus Populations and Larval Habitat Characteristics across the Landscape: Significant Differences Exist between Urban and Rural Land Use Types. Insects. 2021;12(3):196. doi: 10.3390/insects12030196 33668917 PMC7996563

[67] Woolhouse ME, Dye C, Etard JF, Smith T, Charlwood JD, Garnett GP, et al. Heterogeneities in the transmission of infectious agents: implications for the design of control programs. Proc Natl Acad Sci U S A. 1997;94(1):338–42. doi: 10.1073/pnas.94.1.338 8990210 PMC19338

[68] Ellwanger JH, Byrne LB, Chies JAB. Examining the paradox of urban disease ecology by linking the perspectives of Urban One Health and Ecology with Cities. Urban Ecosyst. 2022;25(6):1735–44. doi: 10.1007/s11252-022-01260-5 35855439 PMC9283848

