# Supplementary material for: Environmental and biotic drivers of Aedes albopictus spatiotemporal distribution in a subtropical city: A major transit hub on Argentina’s triple border
Source: PLoS Negl Trop Dis. 2026 Jul 28;20(7):e0013989. doi: 10.1371/journal.pntd.0013989 (PMC13432753; doi:10.1371/journal.pntd.0013989)
Supplement: S1 Table — In both cases, rainfall_30, days active, and Min_T were consistently significant predictors of Aedes albopictus occurrence. Additional variables with strong associations included Road_type, Impervious cover, LST, and environment, while most other predictors were non-significant. See Table 1 for variable definitions. (DOCX) [file pntd.0013989.s003.docx]

| \| **Name** \| **AIC** \| **Significance** \| \| --- \| --- \| --- \| \| rainfall_30 \| 652.37 \| *** \| \| days \| 669.06 \| *** \| \| Min_T \| 669.35 \| *** \| \| Road_type \| 670.44 \| ** \| \| aegbin \| 676.95 \| * \| \| Impervious \| 676.95 \| * \| \| LST \| 677.18 \| * \| \| globhum \| 677.37 \| * \| \| environment \| 678.55 \| NS \| \| globwind \| 679.51 \| NS \| \| oxy \| 679.94 \| NS \| \| month \| 679.98 \| NS \| \| globligh \| 680.22 \| NS \| \| LowVeg \| 680.56 \| NS \| \| age_day \| 680.56 \| NS \| \| globtemp \| 681.13 \| NS \| \| totalpop \| 681.44 \| NS \| \| ph \| 681.63 \| NS \| \| sal \| 681.78 \| NS \| \| CI \| 682.29 \| NS \| \| HgVg \| 682.32 \| NS \| \| NDVI \| 682.62 \| NS \| \| Water_m \| 682.62 \| NS \|   **Model1:albobin~1+(1–quad)+(1–Seasons)** | \| **Name** \| **AIC** \| **Significance** \| \| --- \| --- \| --- \| \| rainfall_30 \| 647.68 \| *** \| \| days \| 665.29 \| *** \| \| environment \| 665.40 \| *** \| \| Impervious \| 667.59 \| *** \| \| Min_T \| 667.73 \| *** \| \| LST \| 672.79 \| ** \| \| Road_type \| 671.97 \| ** \| \| Water_m \| 673.38 \| ** \| \| globhum \| 679.40 \| * \| \| LowVeg \| 679.57 \| * \| \| totalpop \| 678.43 \| NS \| \| month \| 678.38 \| NS \| \| oxy \| 678.44 \| NS \| \| aegbin \| 678.59 \| NS \| \| CI \| 678.7 \| NS \| \| globtemp \| 679.04 \| NS \| \| NDVI \| 679.28 \| NS \| \| ph \| 679.37 \| NS \| \| globlight \| 680.46 \| NS \| \| HgVeg \| 680.49 \| NS \| \| age_day \| 680.54 \| NS \| \| globwind \| 680.56 \| NS \| \| sal \| 681.00 \| NS \|   **Model2:albobin~1+(1–ID)+(1–Seasons)** |
| --- | --- | --- | --- | --- | --- | --- | --- | --- | --- | --- | --- | --- | --- | --- | --- | --- | --- | --- | --- | --- | --- | --- | --- | --- | --- | --- | --- | --- | --- | --- | --- | --- | --- | --- | --- | --- | --- | --- | --- | --- | --- | --- | --- | --- | --- | --- | --- | --- | --- | --- | --- | --- | --- | --- | --- | --- | --- | --- | --- | --- | --- | --- | --- | --- | --- | --- | --- | --- | --- | --- | --- | --- | --- | --- | --- | --- | --- | --- | --- | --- | --- | --- | --- | --- | --- | --- | --- | --- | --- | --- | --- | --- | --- | --- | --- | --- | --- | --- | --- | --- | --- | --- | --- | --- | --- | --- | --- | --- | --- | --- | --- | --- | --- | --- | --- | --- | --- | --- | --- | --- | --- | --- | --- | --- | --- | --- | --- | --- | --- | --- | --- | --- | --- | --- | --- | --- | --- | --- | --- | --- | --- | --- | --- | --- | --- |

Signif.codes:0‘***’0.001‘**’0.01‘*’0.05‘NS’>0.05
